# Unsymmetrical Mesoporphyrinic Complexes of Copper (II) and Zinc (II). Microwave-Assisted Synthesis, Spectral Characterization and Cytotoxicity Evaluation 

**DOI:** 10.3390/molecules16075604

**Published:** 2011-06-30

**Authors:** Rica Boscencu

**Affiliations:** Faculty of Pharmacy, “Carol Davila” University of Medicine and Pharmacy, 6 Traian Vuia St., 020956 Bucharest, Romania; Email: rboscencu@yahoo.com; Tel.: +4021-3111152; Fax: +4021-3111152

**Keywords:** unsymmetrical mesoporphyrinic complexes, microwave irradiation, solvatochromy, cytotoxicity, HT29 cell line

## Abstract

New unsymmetrical mesoporphyrinic complexes, namely 5-(4-hydroxyphenyl)-10,15,20–tris-(4-carboxymethylphenyl)–21,23-Zn(II)-porphine and 5-(4-hydroxyphenyl)-10,15,20–tris-(4-carboxymethylphenyl)–21,23-Cu(II)-porphine, were synthesized using a microwave irradiation method. The structures of the porphyrinic complexes were confirmed using FT-IR, UV–Vis, EPR and NMR spectral data. The spectral absorption and emission properties of the porphyrinic complexes were studied in organic solvents of different polarities and the influence of solvent polarity on the wavelengths of the absorbance and fluorescence band maxima is described. The cytotoxicity evaluation of the porphyrinic complexes was performed on human colon adenocarcinoma cell line HT29 for different doses and incubation times. The obtained result indicates a lack of or low toxicity for both compounds, thus recommending them for further testing in light activation protocols.

## 1. Introduction

Metalloporphyrins have attracted attention for many decades because of their importance in the field of biomedicine, especially in diagnosis and treatment of cancer [1,2,3,4,5,6] as well as in photodynamic antimicrobial therapy [7,8,9,10]. 

Photodynamic therapy as a selective treatment method that involves the administration of a pharmaceutical formulation containing a tetrapyrrolic photosensitizer, its selective localization at the cellular level, followed by generation by irradiation with light in the red region of the visible spectrum of cytotoxic species such as singlet oxygen (^1^O_2_) that destroys the sick cells [11,12,13,14,15]. The development of new candidates for potential application as sensitizers in photodynamic therapy requires of them a high purity and simple production under laboratory conditions, a good localization at cellular and subcellular level, great selectivity for the malignant tissue, photoactivity at wavelengths higher than 600 nm, lack of toxicity in the absence of the exciting light, rapid elimination from the body after the treatment is performed and nontoxic metabolites [16,17,18,19,20,21]. 

Porphyrins and metalloporphyrins have also been investigated for use as fluorescent markers in cancer diagnosis because have appropriate fluorescence characteristics such as convenient emissions in the phototherapeutic window (~600 to 1,100 nm), high singlet oxygen quantum yield, large Stokes shifts, and reduced photolability [1,3,4]. For diagnostic and photodynamic therapeutic applications the biomedical efficiency of the porphyrinic structures is determined by its subcellular localization, which depends on the structural and physicochemical characteristics of the photosensitizer [22]. Therefore, depending on the nature of the peripheric substituents and the metallic ion, photosensitizers can be localized within the mitochondria, lysosomes, endoplasmic reticulum, Golgi apparatus and plasma membranes [23]. Thus, by modifying the charge density and its distribution at the periphery of the tetrapyrrolic macrocycle it is possible to control the route of these compounds to the target cells [24,25]. The purity of these compounds is also very important because the presence of trace amounts of impurities can introduce significant errors in the photodynamic activity. 

**Figure 1:**
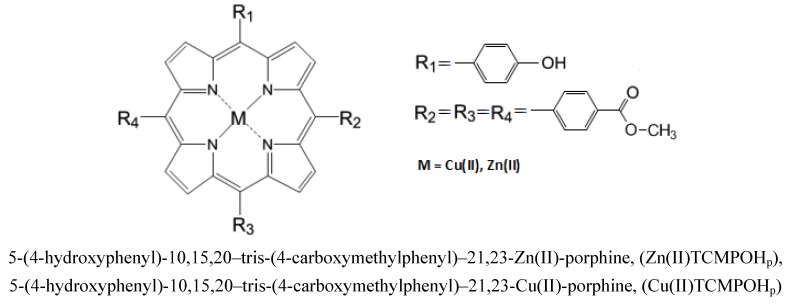
General structures of 5-(4-hydroxyphenyl)-10,15,20–tris-(4-carboxymethyl-phenyl)–21, 23 M(II) porphines.

This paper reports the microwave-assisted synthesis of some new unsymmetrical porphyrinic complexes, namely 5-(4-hydroxyphenyl)-10,15,20–tris-(4-carboxymethylphenyl)–21,23-Zn(II)-porphine (denoted as Zn(II)TCMPOH_p_ - Figure 1) and 5-(4-hydroxyphenyl)-10,15,20–tris-(4-carboxy-methylphenyl)–21,23-Cu(II)-porphine (denoted as Cu(II)TCMPOH_p_ - Figure 1), their spectral properties and cytotoxicity evaluation with a view to assessing their possible use in the photodiagnosis and photodynamic therapy of cancer. 

## 2. Results and Discussion

Microwave assisted synthesis has attracted considerable attention over the last few years and has been applied successfully in the preparation of tetrapyrrolic complexes. The main advantages of synthesis via microwave irradiation are significant reductions in reaction times and side reactions, increased yields, ease of purification and minimization of the amount of solvent used [26,27,28,29,30,31]. For thse these reasons in this paper the synthesis of the porphyrinic complexes was carried out using microwave irradiation assisted synthesis. The synthetic reactions have been successfully repeated several times with identical results and the porphyrinic complexes thus formed were then characterized by elemental analysis, IR, UV–Vis, NMR, EPR spectrometry and evaluated in terms of cytotoxicity using HT29 (human colon adenocarcinoma) cell line for different doses and incubation times.

### 2.1. Infrared Spectra

The most relevant results extracted from the IR spectra of the synthesized unsymmetrical porphyrinic complexes are presented in Table 1. 

**Table 1:** Characteristic IR vibrations of the unsymmetrical porphyrinic complexes.

Characteristic vibration	Wavenumber of the IR band (cm^−1^)	
	Zn(II)TCMPOH_p_	Cu(II)TCMPOH_p_
ν_O-H_	3490 *m*	3489 *m*
ν_C-H_	2919 *m*	2920 *m*
ν_C-H__from -O-CH3_	2850 *m*	2850 *m*
ν_C=O_	1716 *m*	1718 *m*
ν_C-N_	1595 *s*	1596 *s*
ν_C=N_	1451 * m*	1453 * m*
ν_C-H__pyrrole_	1390 *m*	1384 *w*
ν_C-O_	1158 *m*	1157 *m*
δ_C-H_	1029 *m*	1032 *w*
γ_C-C_	859 *w*	857 *w*
γ_C-N__pyrrole_	790 *m*	789 *m*

The intensities of the signals are described as weak *(w)*, medium *(m)*, strong *(s) *and very strong *(v.s.)*.

The IR spectra of the porphyrinic ligand [32] shows a medium band at ~3,310 cm^−1^ assigned to the N-H stretching vibration of the porphyrinic core, which not found in the spectrum of the mesoporphyrinic complexes. This observation supports the coordination of both nitrogen atoms of the porphyrinic core to the metal center. 

The IR spectrum of the synthesized copper and zinc complexes includes typical vibration modes of both porphyrin macrocycle and phenyl substituents. Thus, the medium band at ~2,919 cm^−1^ is attributed to the C–H stretching of the phenyl groups. The bands at 3,490 cm^−1^ are assigned to the O-H stretching vibration of the –OH functional group in the Zn(II)TCMPOH_P_ and Cu(II)TCMPOH_P_. The bands of the complexes in the range of 1,716–1,718 cm^−1^ are assigned to C=O stretching vibrations. In addition, in the IR spectra of the unsymmetric porphyrinic complexes the medium absorption band at about 1,158 cm^−1^ can be attributed to the C-O groups. The IR spectrum of the porphyrinic complexes indicates the presence of the -O-CH_3_ group at 2,850 cm^−1^. A band attributed to ν_C=N_ was present at 1,451–1,453 cm^−1^. In addition, the band at about 1,595 cm^−1^ can be assigned to C-N stretching vibrations and the band at ~1,390 cm^−1^ includes the C–H stretching of the pyrrole ring.

### 2.2. Absorption and Fluorescence Spectra

**Figure 2:**
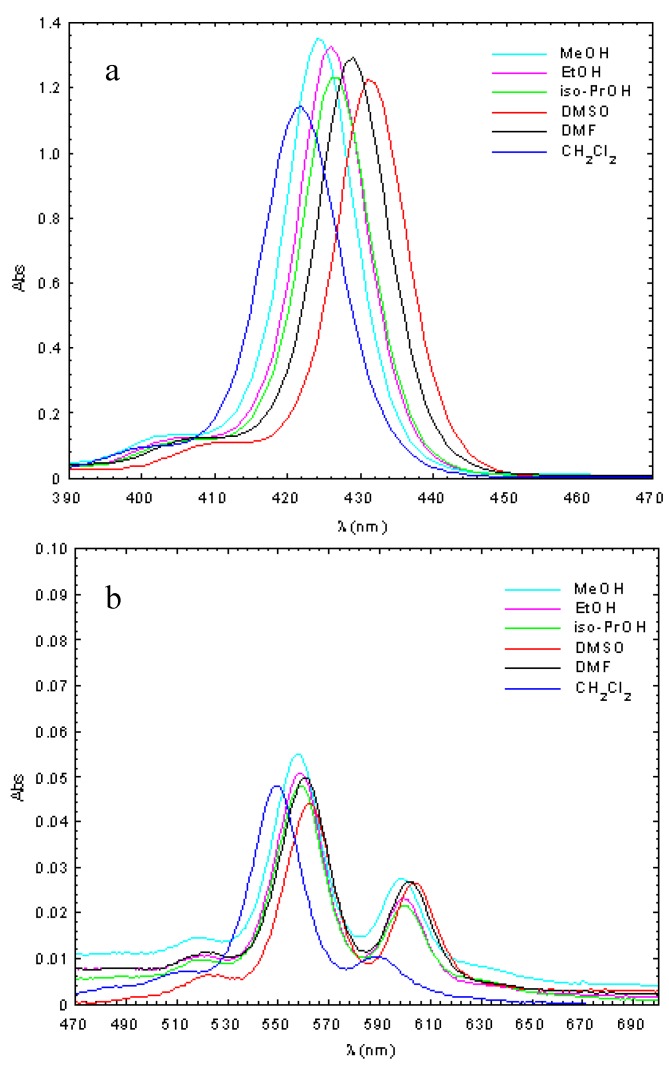
Absorption spectra of 5-(4-hydroxyphenyl)-10,15,20–*tris*-(4-carboxymethyl-phenyl) – 21, 23 Zn(II) porphine in different solvents (c = 2.5 × 10^−6 ^M, **a**-Soret band; **b**-Q bands).

The absorption spectra of Zn(II)TCMPOH_p_ and Cu(II)TCMPOH_p_ were studied in solvents with different polarities (methanol, ethanol, isopropyl alcohol, dimethyl sulfoxide, dimethylformamide, dichloromethane) for solutions containing 2.5 × 10^−6^ M of the porphyrinic complex. The obtained results are presented in Figure 2 and Figure 3 and Table 2.

**Figure 3:**
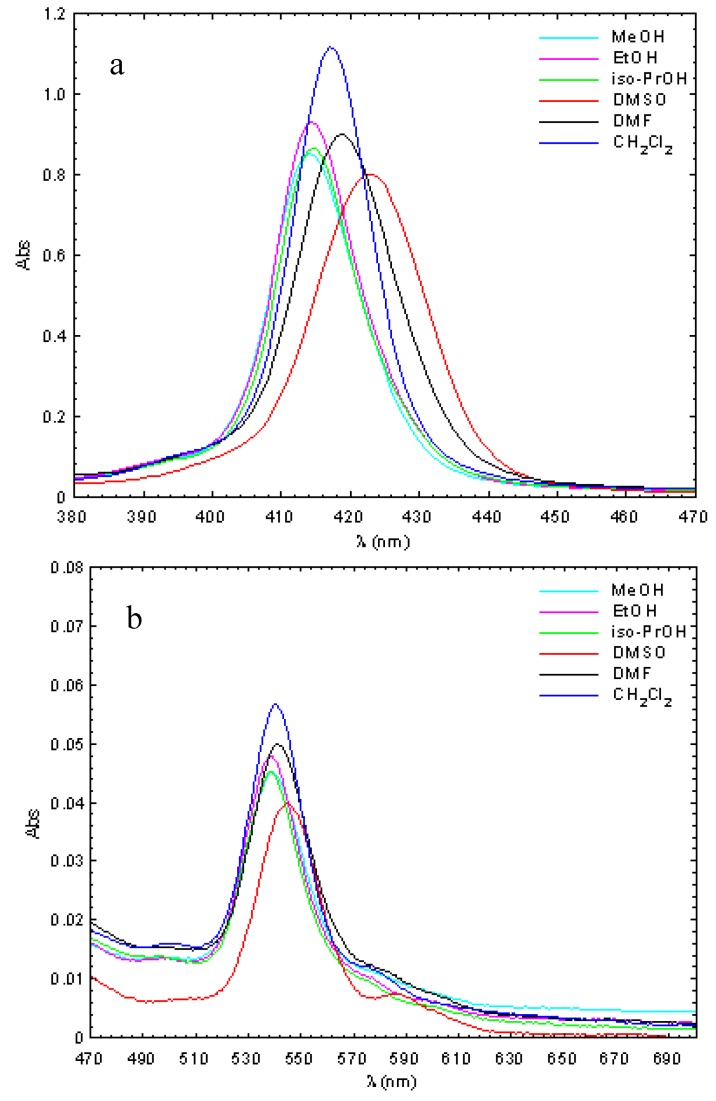
Absorption spectra of 5-(4-hydroxyphenyl)-10,15,20–*tris*-(4-carboxymethyl-phenyl)– 21, 23 Cu(II) porphine in different solvents (c = 2.5 × 10^−6^ M, **a**-Soret band; **b**-Q bands).

**Table 2:** Maximum wavelength and molar absorptivity of the Zn(II)TCMPOH_p_ and Cu(II)TCMPOH_p_ in different solvents (c = 2.5 × 10^−6^ M).

Solvent	λmax (nm) [lgε (L mol^−1^ cm^−1^)]		
	Soret band	Q bands	
	B(0,0)	Q_y_(0,0)	Q_x_(1,0)
*5-(4-hydroxyphenyl)-10, 15, 20–tris-(4-carboxymethylphenyl)–21,23-Zn(II) porphine*			
MeOH	424.5 [5.732]	557.7 [4.342]	598.8 [4.049]
EtOH	426.0 [5.723]	558.6 [4.310]	599.7 [3.964]
iso-PrOH	426.7 [5.693]	558.6 [4.283]	600.0 [4.033]
CH_2_Cl_2_	421.9 [5.659]	549.1 [4.282]	588.6 [3.602]
DMF	428.9 [5.713]	560.7 [4.301]	601.8 [4.033]
DMSO	431.5 [4.954]	562.8 [4.246]	603.6 [4.028]
MeOH	414.2 [5.532]	538.6 [4.260]	-
EtOH	414.5 [5.571]	538.9 [4.283]	-
iso-PrOH	414.8 [5.540]	538.9 [4.259]	-
CH_2_Cl_2_	417.1 [5.649]	540.1 [4.356]	-
DMF	418.8 [5.555]	541.3 [4.298]	578.1(sh)
DMSO	422.9 [5.507]	545.2 [4.203]	587.4 [3.477]

sh – shoulder; MeOH = methanol, EtOH = ethanol, *iso*-PrOH = isopropyl alcohol, DMF = dimethylformamide, CH_2_Cl_2_= dichloromethane, DMSO = dimethyl sulfoxide.

The obtained molecular electronic spectra of the copper and zinc synthesized complexes are typical of metalloporphyrins [33,34,35,36,37]. These consist of the Soret band, as a result of the a_1u_ (π) → e_g_ (π∗) transition and two Q bands bands corresponding to the a_2u_ (π) → e_g_ (π∗) transition.

Thus, the UV–Vis spectra of the Zn(II)TCMPOH_p_ complex exhibited one Soret band in the spectral range of 421–432 nm accompanied by two Q bands, in the 549–563 nm and 588–604 nm spectral range, respectively. The absorption spectra of Cu(II)TCMPOH_p_ display a Soret band in the 414–423 nm wavelength region and either one or two Q bands between 538–590 nm, depending on solvent polarity. 

The main differences observed in the absorption spectra of the two porphyrinic complexes are determined by the nature of the metallic center and solvent polarity. Thus, in the absorption spectra of metalloporphyrinic complexes in various solvents, a blue shift of the spectral bands was observed with increasing solvent polarity [38]. These spectral changes can be ascribed to the formation of proton bridges between the polar solvent molecules and the porphyrinic substitutents due to the polarity-induced and proton properties of the alcohol. Also, from the absorption spectra of copper porphyrin can be observed that the second Q band has much smaller intensity or disappeared with increasing solvent polarity.

Compared with Zn(II)TCMPOH_p,_the spectral bands of the Cu(II)TCMPOH_p_shows a blue shift around 10–12 nm for the Soret band and about 20 nm for Q bands. In agreement with Gouterman’s theory [39], these spectral differences can be explained by stronger conjugation effects for the copper 4p_z_ orbital with the π electrons of the tetrapyrrolic ring, effects that cause a decrease the energy of the a_1u_ (π) and a_2u _(π) orbitals relative to the e_g_ (π∗) orbitals with increased energy available for electron transitions and the blue shift of the spectral bands.

Of the synthesized metalloporphyrins, fluorescence signals were only detected for Zn(II)TCMPOH_p _under the experimental conditions used. The emission spectra of 5-(4-hydroxyphenyl)-10,15,20–tris-(4-carboxymethylphenyl)–21,23-Zn(II) porphine in different solvents, at room temperature, were recorded at λ_ex _= 420 nm. 

The fluorescence spectral data shows for Zn(II)TCMPOH_p_ two bands located in the 605–660 nm spectral region (see Table 3) and reveal smaller shifts of the emmision maxima by changing the solvent polarity.

**Table 3:** The fluorescence data of Zn(II)TCMPOH_p_(c = 2.5 × 10**^−^**^6^M, λ_ex _= 420 nm).

Solvent	λmax (nm) [I_f_] (a.u.)	
	Q_x_(0,0)	Q_x_(0,1)
MeOH	605.2 [379.3]	651.8 [63.2]
EtOH	606.8 [740.2]	651.6 [118.0]
*iso*-PrOH	607.0 [776.8]	653.1 [126.4]
CH_2_Cl_2_	608.9 [585.5]	653.5 [130.7]
DMF	609.7 [490.7]	654.9 [81.5]
DMSO	610.9 [380.8]	656.0 [49.9]

MeOH = methanol, EtOH = ethanol, *iso*-PrOH = isopropyl alcohol, DMF = dimethylformamide, CH_2_Cl_2_= dichloromethane, DMSO = dimethyl sulfoxide.

### 2.3. Dark Cytotoxicity Tests

The short-term cytotoxicity evaluation of a metalloporphyrinic compound designed to be used as a photosensitizer is mandatory for its efficiency validation in antitumor therapy when seeking a new therapeutic agent [40] and of its use in imagistics [41]. As well, a long incubation period with the porphyrinic complexes structures provides the answer to the question concerning the possibility of a delayed cytotoxicity installation in the cells when the phototherapeutic aim is the objective, or on their permanence in the cellular structures when imagistic detection is sought.

In order establish the dark cytotoxicity profile, the new mesoporphyrinic complexes were evaluated *in vitro* using HT29 (human colon adenocarcinoma) cell line for different doses and incubation times. For this purpose the cellular viability and proliferative capacity were assessed upon each round of cell incubation with the synthesized metalloporphyrins. 

Viability assay at short-time incubation stressed the point that at low loading doses (1–5 µM) there are no differences between the two porphyrinic complexes behavior. For doses greater than 5 µM, only Zn(II)TCMPOHp induces an increase in the LDH release by HT29 cells (Figure 3a). The effect of the metallic ion of the porphyrinic complex on the cell viability is highlighted at 24 h of incubation and for concentrations higher than 5 µM. In these conditions, only Cu(II)TCMPOHp has a visible influence on the cell viability (Figure 3b).

**Figure 3:**
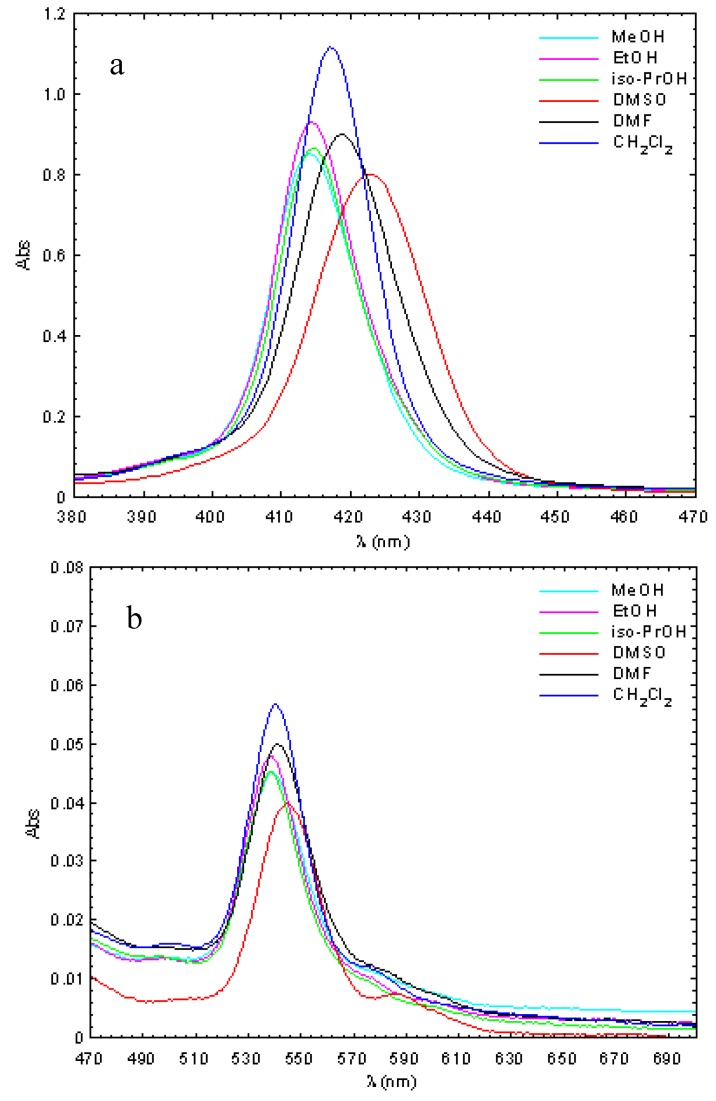
The viability of HT29 cells incubated 2h (**a**) and 24h (**b**) with Zn(II)TCMPOHpand Cu(II)TCMPOHp; the LDH release registered as OD at 490 nm.

**Figure 4:**
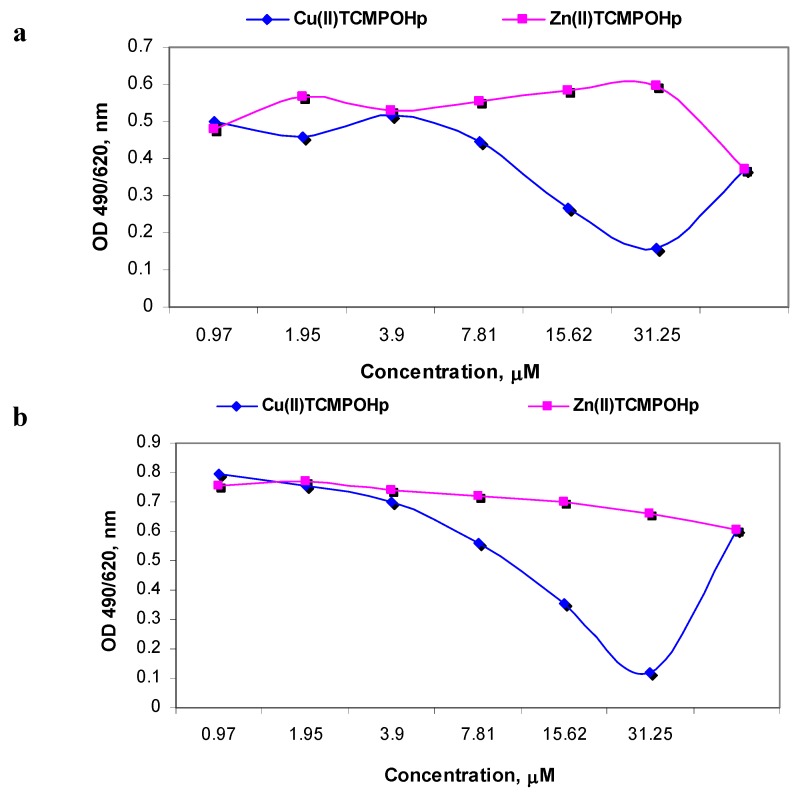
The proliferation of HT29 cells incubated 2 h (**a**) and 24 h (**b**) with Zn(II)TCMPOH_p_ and Cu(II)TCMPOH_p_; the MTS reduction reaction registered as OD at 490/620 nm.

Proliferative capacity assay performed following two hours incubation suggests slight differences between the compounds (Figure 4a). The 3.95 µM concentration results in a homogeneous cell response for both compounds. Cu(II)TCMPOHp inhibits the cellular proliferation only in concentrations higher than 10 µM. Following 24 hours incubation Zn(II)TCMPOHp maintains its nontoxic characteristics related to the proliferative function of the cells, while for Cu(II)TCMPOHp the effect is dose-dependent (Figure 4b). 

## 3. Experimental

### 3.1. Materials and Methods

Commercially available chemicals and solvents were used as received from Sigma-Aldrich and Merck. For the microwave assisted synthesis, a domestic microwave oven was used, with temperature and power control. The elemental analysis of C, H and N was performed with an automatic Carlo Erba L-1108 analyzer. IR spectra were recorded with a FT-IR 400D Nicolet Impact spectrophotometer. The substances under analysis, previously dried for 24 h at 150 °C, were processed as KBr (spectrally pure) pellets. The spectra were recorded in the 4,000–500 cm^−1^ spectral range. The molecular absorption spectra of the mesoporphyrinic complexes were recorded with the use of a Lambda 35 Perkin-Elmer spectrophotometer in different solvents (MeOH, EtOH, *iso*-PrOH, DMF, DMSO, CH_2_Cl_2_) using a 10 mm path length quartz cell, in single beam mode. Fluorescence spectra were recorded with a Jasco FP 6500 spectrofluorimeter. 

The metalloporphyrin solutions were freshly prepared in the spectrally pure solvents at the concentration 2.5 × 10^−6^ M and kept in dark until the measurement to prevent photodegradation. The NMR spectra of the zinc porphyrinic complex were recorded with a 400 MHz Bruker NMR spectrometer. EPR spectra of the copper porphyrinic complex were recorded on powders at room temperature using a ART-6 spectrometer, operating in the X band (9.01 GHz), equipped with a field modulation unit of 100 KHz.

The preliminary toxicological studies in the presence of the unsymmetrical mesoporphyrinic complexes consisted in viability and proliferation studies performed on human colon adenocarcinoma cell line HT29. The HT29 cell line was purchased from the European Collection of Cell Cultures (ECACC, catalog no. 91072201) and cultured in RPMI 1640 medium supplemented with 100 U/mL penicillin, 0.1 mg/mL streptomycin, 0.25 μg/mL amphotericin, 2 mM glutamine and 10% fetal bovine serum. The cells were maintained at 37 °C in a 5% CO_2_ humid atmosphere for standard cultivation and during all the toxicological tests.

The porphyrinic complexes solutions were handled under sterile conditions. Ten mM stock solutions of the compounds to be tested were prepared in DMSO by sonication at 22,000 Hz for 30 seconds. For the toxicological tests the solutions were further diluted in RPMI 1640 culture medium for the toxicological tests in the range 0.97–31.25 µM. 

For the viability and the proliferation test assay, the cells (cell density 5 × 10^−4^ cells/mL) were incubated with each of the tested concentrations for 2 and 24 h. A cellular control consisting in unloaded/untreated HT29 cells was used for every replicate of the tests, corresponding to each incubation time. 

For cellular viability we have used lactate dehydrogenase (LDH) release test [42], using CytoTox 96® Non-Radioactive Cytotoxicity Test (Promega). For testing cell proliferation by means of the tetrazolium salt (MTS) reduction test [43], we have used CellTiter 96® AQeous One Solution Cell Proliferation Assay kit (Promega). Briefly, at end of each incubation time with the mesoporphyrinic complexes, the cell supernatants were collected for LDH test whilst the cell sediment was used for MTS assay. Results were expressed as triplicate’s mean of optical density (OD) ± SD, recorded on a Jasco V630 spectrophotometer, in a single beam mode, at 490 nm without any reference (for LDH release in the cell viability test) and at 490 nm with reference at 640 nm (for MTS reduction test).

### 3.2. Synthesis of 5-(4-hydroxyphenyl)-10, 15, 20–tris-(4-carboxymethylphenyl)–21, 23-Zn(II)-porphine (Zn(II)TCMPOH_P_)

A mixture of 4-hydroxybenzaldehyde (1.12 g, 0.01 mol), methyl 4-formyl benzoate (4.92 g, 0.03 mol), freshly distilled pyrrole (2.76 mL, 0.04 mol), anhydrous zinc chloride (1.36 g, 0.01 mol) and 2–3 g of silica gel 60 (200–500 μm, 35–70 mesh) in the presence of 2,6-dimethylpyridine (1 mL) was irradiated in a Pyrex bottle with a microwave oven set at 175 °C, 450 W for 10 min. Extraction of samples for monitoring the synthesis by UV–Vis spectroscopy was performed after every 2 min of irradiation.

The crude product was first dissolved in dichloromethane, then filtered and finally purified on a chromatography column by repeated elution, using silica gel (100–200 mesh size) as stationary phase and dichloromethane as eluent. The solution of the zinc porphyrinic complex was concentrated by simple distillation. The obtained violet crystals were dried at ≈ 100 °C for 12 h. The compound of interest was obtained with a yield of about 48%. Elemental analysis for C_50_H_34_N_4_O_7_Zn: calculated C 69.2, H 3.92, N 6.45; found C 69.04, H 3.80, N 6.28. ^1^H-NMR: δ_H_(400 MHz, CDCl_3_), ppm: 4.13 (9H, s, O-CH_3_), 6.5 (1H, s, brs.,OH), 8.00 (2H, d, H*_metha-Ph-OH_*), 8.2 (2H, d, H*_ortho-Ph-OH_*), 8.3 (6H, d, H*_ metha-Ph-COOCH3_*), 8.4 (6H, d, H*_ ortho-Ph-COOCH3_*), 8.9 (6H, d, H_βpyrr2_), 9.0 (2H, d, H_βpyrr3_).

The comparative study of the ^1^H-NMR data of the unsymmetrical mesoporphyrinic ligand and its zinc complex reveals the absence of the signal at −2.78 (2H, s, N-H) [32], indicating the involvement of both nitrogen atoms of porphyrinic ring in the coordination after deprotonation. Other signals in the NMR spectrum of the complex are similar to those of the ligand.

### 3.3. Synthesis of 5-(4-hydroxyphenyl)-10, 15, 20–tris-(4-carboxymethylphenyl)–21, 23-Cu(II)-porphine (Cu(II)TCMPOH_P_)

A mixture of anhydrous CuCl_2_ (1.34 g, 0.01 mol), methyl 4-formyl benzoate (4.92 g, 0.03 mol), 4-hydroxybenzaldehyde (1.12 g, 0.01 mol), freshly distilled pyrrole (2.76 mL, 0.04 moli), 2–3 g of silica gel 60 (200–500 μm, 35–70 mesh) and 2,6-dimethylpyridine (1 mL) was irradiated in a microwave oven at 180 °C, 475 W, for 8 min. The extent of the complexation reaction was monitored by UV–Vis spectroscopy and for this purpose extraction of samples was performed after every 2 min of irradiation.

The product was dissolved in dichloromethane, filtered and then purified on a chromatography column. The solid product was chromatographed on silica gel (100–200 mesh size) with dichloromethane as eluent. The solution of the copper porphyrinic complex was concentrated by simple distillation. The obtained dark red crystals were dried at ≈ 100 °C for 12 h. 

The copper unsymmetrical porphyrinic complexes were obtained with a yield of about 52%. Elemental analysis for C_50_H_34_N_4_O_7_Cu: calculated C 69.32, H 3.93, N 6.47; found C 69.19, H 3.82, N 6.36. The EPR parameters are: g_||_=2.172, g_⊥_=2.050, A_||_= 202G, A_⊥_=30 G. These values are in good agreement with literature data and confirm a square planar geometrical arrangement of nitrogen atoms around the copper ion [44,45]. Also, the g_||_ value (g_||_ < 2.3) indicates a covalent character of the Cu-N bonds in the copper-porphyrinic complex [46].

## 4. Conclusions

The paper describes the microwave irradiation assisted synthesis, spectral properties and preliminary cytotoxicity investigation of some new unsymmetrical porphyrinic complexes, namely 5-(4-hydroxyphenyl)-10,15,20–tris-(4-carboxymethylphenyl)–21,23-Zn(II)-porphine and 5-(4-hydroxy-phenyl)-10, 15, 20–tris-(4-carboxymethylphenyl)–21,23-Cu(II)-porphine. These complexes were synthesized for use as active substances in the photodiagnosis and photodynamic therapy of cancer. 

The syntheses of the copper and zinc porphyrinic complexes were confirmed by FT-IR, UV–Vis, EPR and NMR analysis. The influence of environment polarity on the wavelengths of the absorbance and fluorescence band maxima is also described. The spectral changes that occur by increasing environmental polarity can be ascribed to the physical interaction between the porphyrinic substituents and the solvent molecules.

Both tetrapyrrolic complexes were evaluated in terms of cytotoxicity using HT29 (human colon adenocarcinoma) cell line for different doses and incubation times. The results showed no significant toxicity on the investigated cell line and suggest that these novel complexes are promising active substances for the photodiagnosis and photodynamic therapy use.
